# Correlation between the Neutrophil-to-Lymphocyte Ratio and Multiple Sclerosis: Recent Understanding and Potential Application Perspectives

**DOI:** 10.1155/2022/3265029

**Published:** 2022-10-26

**Authors:** Qingqing Zhou, Rui Jia, Jingxia Dang

**Affiliations:** Department of Neurology, The First Affiliated Hospital of Xi'an Jiaotong University, Xi'an, China

## Abstract

Multiple sclerosis (MS) is a chronic debilitating immune-mediated disease of the central nervous system, which causes demyelination and neuroaxonal damage. Low-grade systemic inflammation has been considered to lead to pathogenesis owing to the amplification of pathogenic immune response activation. However, there is a shortage of reliable systemic inflammatory biomarkers to predict the disease activity and progression of MS. In MS patients, a series of cytokines and chemokines promote the proliferation of neutrophils and lymphocytes and their transfer to the central nervous system. The neutrophil-to-lymphocyte ratio (NLR), which combines the information of the inherent and adaptive parts of the immune system, represents a reliable measure of the inflammatory burden. In this review, we aimed to discuss the inflammatory response in MS, mainly the function of lymphocytes and neutrophils, which can be implemented in the utility of NLR as a diagnostic tool in MS patients. The underlying pathophysiology is highlighted to identify new potential targets for neuroprotection and to develop novel therapeutic strategies.

## 1. Introduction

Multiple sclerosis (MS) is a neuroinflammatory and neurodegenerative disease that can lead to focal lesions of the brain and spinal cord. It is characterized by focal demyelinated lesions, segmental save of axons, and glial scar hyperplasia [1,2]. The MS pathological process includes the breakdown of the blood-brain barrier (BBB), inflammatory infiltration, microglia activation, demyelination, oligodendrocyte loss, gliosis, and axonal degeneration [3]. Central nervous system (CNS) inflammation is a major driver of MS disease pathogenesis [4]. It is generally believed that MS is multifactorial, and both environmental and genetic factors seem to be involved in MS [5]. The genome-wide association study (GWAS) has identified more than 100 genetic variants associated with MS, which are mainly locked to the adaptive immune system [5,6]. As for the pathogenesis, T lymphocytes play major roles in guiding immune response, which could trigger and adjust the entrance and transfer of inflammatory cells to the CNS. In addition, more and more evidence shows that B cells play an element role in the pathogenesis and progression of MS [7–9].

The main causes of chronic inflammation are macrophages, lymphocytes, and plasma cell infiltration [10]. Correspondingly, neutrophils and macrophage cells migrate to inflammatory areas via chemokines and cytokines which are responsible for acute inflammation [11]. In line with the deepened understanding of the potential pathophysiological mechanisms of MS, the therapeutics for lymphocytes make great progress. Accordingly, the increase in neutrophil count is usually related to the occurrence and severity of inflammation [12–14]. As an inflammatory marker, the ratio of neutrophils to lymphocytes (NLR) integrates the information from two leukocytes, avoiding the disadvantage of possible infection or other abnormal effects, and has higher clinical significance than other single inflammatory cells [15]. NLR is a parameter reflecting inflammatory index and a predictor of cardiovascular disease, pancreatitis, tumor, and other diseases [16–19]. At present, several studies have shown that NLR can predict the prognosis with neurodegenerative diseases [20–22]. The purpose of this review is to summarize the existing evidence on the relationship between the NLR and MS and evaluate whether the NLR can be used to predict outcomes for clinical management. We will also have an in-depth understanding of the underlying pathophysiological mechanisms, discuss the limitations of current research, and make recommendations for future research.

## 2. Neutrophil-to-Lymphocyte Ratio and MS: Looking Insight Pathophysiology

### 2.1. Neuroinflammation in MS

MS is a multifactorial progressive disorder characterized by multifocal demyelination and perivascular inflammation within the CNS [23,24]. Neuroinflammation is a prominent feature of numerous neurological disorders including MS [25]. Based on the inflammatory nature, immune response targeted therapy is the most widely used treatment. Neuroinflammation is a defense mechanism that initially protects the brain by removing or inhibiting diverse pathogens. This defense against inflammatory response can promote tissue repair and remove cell debris. However, the presence of the persistent neuroinflammatory response is deleterious [26].

The intact endothelium, epithelium, and glial brain barrier together separate the CNS from the periphery, while in neuroinflammatory diseases, the integrality is damaged. The driving force of pathognomonic demyelinating lesions of MS is an autoimmune inflammatory response. MS targets myelin antigens of CNS, involving CD4 + and CD8 + cells, and also, during progressive MS, the role of B cells appears to be prominent, particularly in the context of meningeal inflammation, the formation of ectopic germinal centers, including B cells, was found in the meninges of patients with progressive MS, indicating that the adaptive immune system plays an important role in the pathogenesis [27].The inflammatory response in MS is the cumulative effect of a series of factors, their mediators, and effector molecules, such as cytokines and antibodies [28]. In acute and relapsing MS, the BBB is damaged and becomes leaky, and T cells and B cells invade white matter locally, resulting in typical active demyelinating plaques [2]. Lymphocyte invasion is related to the activity of cytokines in the CNS, while the high expression of cytokines further leads to an increase in disease activity [9]. In addition, the inflammatory cell infiltration composed of CD8 + T cells and B cells is mainly located in the pia matter, which may form a complex aggregation similar to tertiary lymphoid follicles [29]. In progressive MS, there creates a microenvironment in the CNS, which is conducive to the homing and retention of inflammatory cells, and it eventually leads to the basic ineffectiveness of disease modification therapy [30].

Experimental autoimmune encephalomyelitis (EAE) is regarded as a relatively appropriate animal model of MS. It is often used to study the molecular mechanisms of inflammation and neurodegenerative diseases. [31]. Regulatory T (Treg) cell function has been shown to directly influence the ability of mice to induce EAE [32]. The course of EAE is characterized by the infiltration of inflammatory T cells in the CNS [33]. EAE is mainly induced by the proliferation and activity of CNS antigenic specificity CD4+ cells [33,34]. In the EAE model, B cells, as antigen-presenting cells, can interact with CD4+ T cells and initiate an adaptive immune response to produce an inflammatory effect on myelin antigen [35].

The initial protective inflammatory response eventually leads to demyelination and neurodegeneration. A better study of the inflammatory response is of great significance for the treatment of diseases. At present, some established biomarkers are helpful for the diagnosis and prognosis of MS [36]. Biomarkers of MS mainly come from the fields of immunology and neurobiology [37]. These markers include oligoclonal bands, IgG index, anti-AQP-4 antibodies, neurofilaments, and chitinase-3-like-1 [36]. Finding more sensitive inflammatory markers of MS disease is very important for early diagnosis of the disease.

### 2.2. Lymphocytes as Key Inflammatory Cells in MS

The characteristic active demyelinating lesions of the brain and spinal cord in patients with MS are associated with inflammation around blood vessels and brain parenchyma cell infiltration, which is composed of T and B cells [38]. In MS patients, T cells are activated in the peripheral, and then they penetrate the CNS, trigger a central immune response, and further self-maintenance, leading to the myelin sheath and axon damage [39]. T cells in MS lesions express cytotoxic effector phenotypes, mainly CD8+ effector memory T cells (TEM), indicating local antigen stimulation [40]. Although MS was once considered a T-cell-mediated disease, the significant efficacy of rituximab and other similar therapies on MS shows that B cells also play an important role in the pathogenesis of MS [25]. Like T cells, B cells also have proinflammatory and anti-inflammatory subsets. In relapsing MS, T cells are the pathogenic cells, while B cells are the main antigen-presenting cells. On the other hand, in progressive MS, B cells can enhance the conditioned response of the CNS through lymphoid follicles and secretory factors [41]. The intrathecal synthesis of immunoglobulin reflects the clonal expansion of B lymphocytes and plasma cells.

In MS patients, B and T cells interact in the periphery and CNS to contribute to disease pathogenesis [42]. Epstein–Barr virus (EBV) infection suggests a risk factor for MS [43,44]. In MS patient secondary lymphoid organs, due to the deficiency of B cell tolerance, EBV-infected B cells escape the inhibition of CD8+ and T regulatory cells [45]. These activated B cells enter the germinal center and interact with follicular helper T cells to differentiate into pathogenic memory B cells [46]. Under the influence of interferon gamma (IFN-*γ*) and interleukin (IL)-21, B cells develop into memory cells, which in turn activate Th effector cells such as Th17 [47]. Within the CNS, IFN-*γ* and granulocyte-macrophage colony-stimulating factor (GM-CSF) producing T cells and memory B cells probably contact follicle-like structures, promoting CNS inflammation and demyelination [48]. Memory B cells further amplify and differentiate into plasma cells of endocrine antibodies of the CNS to secrete a large number of potentially harmful antibodies, namely, oligoclonal bands [42].

The interaction between B cells and T cells is the central feature of MS pathogenesis [49]. CD8+ T cells and CD20+ B cells dominate in the pathogenesis of all disease stages in MS [27]. CD8+ T cells recognize the endogenous antigenic peptides presented by MHC class I and differentiate into cytotoxic T cells after activation [50]. In active MS lesions, the activation of astrocytes, oligodendrocytes, and axons gradually upregulated the expression of MHC class I, making these cells potential targets for CD8+ T cells in the disease course [34]. CD8+ T cells also have the characteristics of memory cells resident in tissues. Next to T cells, B cell lineage contributes to adaptive immune inflammation of MS patients [25]. In addition, B cells can produce a variety of anti-inflammatory cytokines, such as transforming growth factor-*β*1, IL-35, and IL-10 [51]. In the active lesions of MS, these cells may be reactivated locally, and B cells gradually transform into plasma cells partially. This can be confirmed by the discovery of clonally amplified B cells in cerebrospinal fluid, meninges, and brain parenchyma of MS patients [9]. B cells can pass through the BBB and form ectopic germinal centers in the CNS, and the functions are independent of the periphery [52]. This has coincided with the observation of immunoglobulin synthesis in the CNS of MS patients [34]. In vivo antigen-activated B cells can be used as effective antigen-presenting cells (APCs) to promote the development of MS [53]. Peripheral blood B cells can raise the secretion of numerous inflammatory factors, such as IL-6, lymphotoxin- *α* (LT- *α*), tumor necrosis factor (TNF), and the GM-CSF [54]. CD20+ B cells were particularly numerous in patients with acute MS, as the main component in the early stage of the disease [55]; in contrast, the numbers of plasma cells were significantly higher in lesions from patients with the disease progress [34,56]. B cells may impact MS through a variety of mechanisms, including the establishment of ectopic lymphoid follicles within the CNS, presentation of antigens to T cells, cytokine/chemokine secretion, and autoantibody production in the CNS [42]. In recent years, the essential role of B cells for MS has been validated by successful clinical trials that use anti-CD20 therapy to deplete B cells [7]. B cells may be important target cells to guide the treatment of MS.

The interaction between B cells and T cells is an important driving factor in the pathogenesis of MS. Cytokine production, costimulation, and antigen presentation may contribute to the development of pathogenic B and T cells entering the CNS [27]. This mechanism may be affected by the interaction between genetic and environmental risk factors. The major HLA-DRB1#1501 variants have been shown to promote B cell-mediated induction of T helper (Th) cells in MS patients [57]. A number of identified genetic risk locis, including HLA-DRB1#1501, seem to enhance the B and Th cells [57]. In addition, infectious factors may change the function and reactivity of MS such as EBV as mentioned previously, and several theories have been proposed about how EBV influences MS pathogens [58]. In MS autopsy cases, B cell infiltration was also found around the blood vessels associated with active white matter lesions [34]. The role of these perivascular B cells is to reactivate proinflammatory CD4+ and CD8+ T cells, leading to MS inflammatory response and demyelination [59].

However, the lymphocyte count in the peripheral blood of MS patients may not be significantly increased [60]. T cells, at least in the part degree of B cells, are markers of the disease process and damage activity. The higher the disease activity is, the more these cells are in the tissue. However, there are regional differences in the distribution of these cells [30]. In the late stage of progressive MS, inflammation composed of T and B cells may drop to the level of the age-matched control group [38]. Some studies have also shown that MS patients may have decreased lymphocytes in peripheral blood before treatment [61]. A part of the reason may be the high migration rate of lymphocytes to the CNS, which leads to the increase in the NLR ratio. Moreover, the difference in bone marrow function and the production rate of different immune cells in MS patients may also lead to NLR changes in MS [52,62]. There is also a theory that psychological stress caused by nervous system disorders in MS patients may change the balance between innate immunity and adaptive immunity, resulting in an increase in NLR [63].

### 2.3. Neutrophil Alterations in MS

Previous studies suggested that neutrophils are simple phagocytes of the innate immune system, but the current view is that neutrophils are important effectors and regulatory circuits that control the quantity and quality of immune response [64]. So far, the role of neutrophils in the pathogenesis of neuroinflammation has become more and more attractive [65]. Neutrophil infiltration in the CNS of MS patients may be an early trigger factor of inflammation-causing BBB injury [66]. Neutrophils can secrete a series of cytokines that can influence MS and EAE. These cytokines, such as TNF-*α*, IL-6, IL-12, IL-1*β,* and IFN-*γ*, are considered to have contributed to the cascade of inflammation in the CNS [67].

The concentrations of neutrophil-activated chemokines and neutrophil-derived enzymes in the blood of MS patients were higher than those in control, and these molecules were related to the formation of new inflammatory lesions, and these included CXC chemokine ligand-1 (CXCL1), CXCL8, and myeloperoxidase (MPO) [13]. This cytotoxic effect may involve the secretion of cytokines ROS and matrix metalloproteinase 9 (MMP9). In EAE models, neutrophils may excite cell aggregation and migration by increasing the permeability of the glial cell membrane [24]. In addition, in progressive MS patients, BBB leakage is related to the increased abundance and activity of MMP9 in serum and cerebrospinal fluid (CSF). Furthermore, larger numbers of neutrophil extracellular traps (NETs) were found in the blood of some MS patients, supporting the role of neutrophils in MS pathogenesis [24].

## 3. NLR and Clinical Outcome with MS: The Evidence from Clinical Studies

### 3.1. The Potential Utility of the NLR in MS

In the search of handy accessible biomarkers available for diagnosing MS and forecasting the disease course, several inflammation-related blood biomarkers have been studied, such as TNFa and IL-6, but none proved clinically helpful [74]. For a long time, people have been seeking an inflammatory marker that can divide MS subtypes and forecast disease activity. The discovery of NLR as a biomarker for several diseases can better reflect systemic inflammation than neutrophils or lymphocytes alone [15]. In particular, the NLR has been studied more and more as a marker of systemic inflammation, especially considering its rapid, extensive, and relatively economic evaluation [20]. NLR is related to the disease activity of numerous autoimmune diseases, such as inflammatory bowel disease [75], Sjogren's syndrome [76], rheumatoid arthritis [77], and ankylosing spondylitis [78]. The predictive value of NLR exceeds the neutrophil count alone, and NLR may serve as an inexpensive and easily available supplemental marker in MS [79].

### 3.2. The NLR as a Clinical Tool in the Diagnosis and Prediction of MS

Demirci and colleagues were the first to explore NLR and MS; they analyzed NLR in 102 patients with relapsing-remitting MS (RRMS) and 56 healthy controls (HCs). NLR values were higher in RRMS patients compared with HCs. This study shows that raised NLR can not only differentiate between MS and HCs but also associate with the severity of clinical symptoms [80] (Table 1). Several following studies confirmed similar findings (Table 1). Another study shows that NLR was higher in MS patients compared to HC, indicating the presence of an inflammatory response (Table 1). However, the NLR can only be used as a “supplementary” mark and cannot be used as a diagnostic marker of MS activity alone [79]. The advantage of this study is that the sample size of each stage in MS patients is large and is not affected by other confounding factors [79].

The study by Al Hussain investigated 60 MS patients and 60 HCs and found higher NLR values in MS compared with HCs. However, the confounding factor data were not studied, so the outcome must be interpreted carefully [81] (Table 1). Another large study by Hasselbalch et al. investigated 740 patients with early MS and 1420 HCs and found higher NLR values in MS patients compared with HCs. The study predicted a critical value of 2.07 for MS diagnostic ability [82] (Table 1). This study has two merits: firstly, the sample capacity of this study is large; secondly, the patients have a blood test before taking DMT for the first time, so it has no effect on the drug. Another study shows that high NLRs may contribute to the worsened outcomes reported in MS patients [83] The study by Akil et al. investigated the levels of RRMS patients and NLR levels and showed that the NLR was significantly higher in the RRMS group than in the HCs [84], while there was no relevance between the disease duration, EDSS score, and MRI lesion [84] (Table 1).

The disease activity of MS is characterized by clinical recurrence, new T2 lesions, or GD enhanced lesions. In addition, NLR appears to be able to forecast the demand for remedies promoting the DMT from first-line to second-line, so NLR can be deemed as a substitutable indicator of disease activity. The studies on NLR and disease activity mostly do not include the patients with DMT within 6 months to avoid impact on results. D'Amico et al. found that higher NLR raised the risk of disease activity but did not include data on concomitant diseases or smoking conditions that may affect cell counts. So, the conclusions must be explained carefully [85] (Table 1). Hemond et al. found that NLR was closely associated with increased disability, which was assessed by the expanded disability status scale (EDSS), hence distinguishing the course of progressive and recurrent diseases. This study indicates that the increase in NLR reflects the supplementary and independent marker of MS-related neurological dysfunction and the severity of MRI results [83]. A retrospective study by Guzel et al. found NLR levels to be higher in MS patients with EDSS ≥5 compared with EDSS <5. NLR value may have the ability to distinguish adverse clinical results, with a cut-off value of 4.52. However, the difference in the use of DMT between the two groups makes the conclusion not universal [86] (Table 1). Due to the different action mechanism of DMT in the treatment of MS, some DMTs are more easily to lead to lymphopenia, lymphocytosis, and neutropenia. Therefore, the impact of MDT on the results must be considered. Yetkin and Mirza discuss the NLR in 270 MS patients with treatment naïve relapsing onset and suggest that baseline NLR in primary MS may be conducive to the risk group stratification and the choice of disease-modifying therapies [87] (Table 1). A new study by Gelibter confirms the role of NLR in a cohort of newly diagnosed MS and clinically isolated syndrome (CIS) patients, and the results do not support the NLR as a biomarker of disease activity and disability in patients with MS [88] (Table 1).

Due to the close relationship between the immune system and the pathophysiology of MS, the parameters that can reflect the inflammatory response may be related to the disease process. Obviously, the specificity of NLR is not enough as the final definitive diagnostic instrument of MS. However, it has certain sensitivity and can play a role as a screening tool, which can be used to stratify patients before more invasive or expensive inspection means. Moreover, it can also guide drug selection. Therefore, in order to become a successful screening tool, its sensitivity should be further improved. It may be reasonable to adopt different cut-off values or choose to combine them with other validated biomarkers.

### 3.3. Limitation

There are many uncertainties in the assessment of NLR as a prognostic marker and an efficacy predication for MS. The conclusion of clinical research may overevaluate the relevance and effect and clinical relationship between NLR and MS to a certain extent because the influence of confounding factors is unavoidable. In addition, since NLR is a dynamic index, most of the samples in the study were conducted at the time of admission, which may be single-faceted to a certain degree. Because the presence of symptomatic MRI positive lesions may affect the NRL level, imaging data should be included in the study analysis, while most studies lack MRI MRI-related disease outcomes. Undoubtedly, further polycentric, large sample, forward-looking, standardization studies are needed to identify the comprehensive mechanisms and association between the NLR and MS.

## 4. Conclusions

Overall, comprehending whether changes in peripheral inflammation can affect disease progression may be helpful to obtain a better understanding of the latent pathophysiology of MS. Furthermore, large prospective studies are needed to investigate the correlation between NLR and MS disease progression. Moreover, MS is a tardy progressive disease, and it entails longer follow-up which may be necessary to accurately assess the utility of a biological marker in detecting its onset and determining its severity, including NLR. Also, assessing the changes in the ratios between specific subtypes of neutrophils and lymphocytes could offer a higher sensitivity and/or specificity marker of MS severity and disease progression. This study we summarize existing evidence on the relationship between the NLR and MS and evaluate whether the NLR can be used to predict outcomes and as an effective biomarker for clinical management. We provide insights into the underlying pathophysiological mechanisms and discuss the limitations of the current studies to make recommendations for future research. Further prospective studies are needed to investigate the relationship between NLR and MS. Despite the complexity of neuroinflammation and the lack of existing research, regulating the levels of lymphocytes and neutrophils and related signal pathways may provide clinical interventions for MS.

## Figures and Tables

**Table 1:** Studies investigating the neutrophil-to-lymphocyte ratio NLR in patients with MS.

Study	Study design	MS	HC	NLR cut-off	Sensitivity (%)	Specificity (%)	Findings/conclusions	Confounders addressed^a^	Strengths and/or limitations
Demirci, 2016	Case control	102	56	2.04	81	62.5	NLR values were higher in MS compared with HC, and patients in relapse had higher NLR values than HC and patients in remission. Patients in remission had higher NLR values than HC. High NLR was an independent predictor of disability progression with EDSS >3 as the response variable.	DMT Smoking Conc. Dis.	All patients were nonsmokers and untreated for at least six months prior to inclusion. However, no data on BMI were provided.
Guzel, 2016	Retrospective cross-sectional	127	—	4.52	96.1	57.1	NLR values were higher in patients with EDSS >5 compared with patients with EDSS ≤5. The EDSS score had a weak to moderate correlation with NLR.	DMT Conc. Dis.	The use of DMTs differed between the two EDSS stratified groups and they did not include data on BMI, smoking, or relapse status.
Bisgaard, 2017	Case control	219 and CIS: 19, ON: 140	813	—	—	—	NLR values were higher in MS compared with HC, and higher in patients in relapse than remission. NLR values did not predict an EDSS score ≥4.0.	DMT Smoking Conc. Dis.	The advantage of this study is that the sample size of each stage in MS patients is large and is not affected by other confounding factors.
Al-Hussain,2017	Case control	60	60	—	—	—	NLR values were higher in MS compared with HC. NLR values were correlated with stress scores.		Did not report any data on confounding factors and these results should be interpreted carefully.
Hasselbalch, 2018	Case control	740	1420	2.07	49	70	NLR values were higher in MS compared with HC. NLR values correlated weakly with MSSS.	DMT BMI Smoking	The sample capacity of this study is large; the patients have a blood test before taking DMT for the first time, so it has no effect on the drug.
D'Amico E, 2019	Retrospective, observational	84	—	—	—	—	NLR values were higher in the “low disease activity” group compared with the “high activity group.” High activity was defined as (≥ 2 relapses in the year prior to study entry and (≥ 1 gadolinium-enhancing lesion at the time of the study. No associations were observed between NLR values and patient characteristics (gender, age, and EDSS at onset).	DMT Conc. Dis.	The sample size is small. No data regarding concomitant diseases that could alter the neutrophil count or other data such as smoking status.
Hemond, 2019	Cohort study, retrospective analysis	483	—	2.1	—	—	NLR values were higher for MS patients on cyclophosphamide and fingolimod treatment and significantly lower when treated with interferons and natalizumab. Higher NLR values were associated with increased EDSS scores, and PMS was compared with RRMS. Increased NLR values were associated with higher depression (CES-D) scores, higher fatigue scores (MFIS), and lower physical quality of life (SF-36).	DMT BMI Smoking	This study includes a large and well-characterized sample of MS patients, including self-reported psychological outcome measures, specific DMT use, and quantitative neuroimaging pathology, while the results are of an associative nature only, and thus no conclusions should be drawn regarding causality.
Goldman D, 2020	Retrospective, observational	103	—	2.0	—	—	At baseline, the NLR values were 2.5 ± 1.9 for all included patients. Over 50% of MS patients had high NLR (defined as NLR >2). In the follow-up samples, after starting treatment with dimethyl fumarate, the NLR values were 3.6 ± 2.5.	DMT BMI Conc. Dis.	This study considered dimethyl fumarate-associated neutropenia. The limitations of this study reflect its retrospective nature.
Yetkin MF,2020	Cohort study, retrospective Analysis	270	—	2.365	56.94	60.1	NLR values were higher in MS patients who had breakthrough disease activity, defined as the escalation group (median (min-max): 2.5 (0.1–13.8)) compared with the nonescalation group (median (min-max): 2.1 (0.6–12.8)).	DMT Smoking Cont. Dis.	The strengths of this study are the number of subjects and long-term follow-up, while not having detailed information about the comorbid status and lifestyle of the patients.
Gelibter, 2021	Cohort study, retrospective analysis	121	—	—	—	—	Association was found neither between NLR and disease activity nor with other clinical measures.	DMT BMI Smoking	This study compared the validity of NLR with established markers, such as serum neurofilament light chain (nf). CSF microvesicles (CSF-MVs) and CSF IgG indices. The NLR was also investigated in relation to the brain and spinal cord MRI findings.
Akil, Alp, 2021	Case- control	83	44	—	—	—	The NLR levels were found to be significantly higher in the patient group than in the HC group. There was also a significant difference between the serum NLR levels of the relapse and HC.	Smoking	The NLR levels of the RRMS patients were measured while in relapse, 1 month after relapse, and while in remission. Moreover, the patients were grouped based on the lesion burden of MRI, while the sample size was limited.

Abbreviations: HC: healthy control, MS: multiple sclerosis, MSSS: multiple sclerosis severity scale, NLR: neutrophil-to-lymphocyte ratio, ON: optic neuritis, PMS: progressive MS, RRMS: relapse-remitting MS, SF-36: short form 36, and EDSS: expanded disability status scale. Articles were screened for following confounders: smoking history, BMI, information on disease-modifying therapy (DMT), and concomitant diseases (Conc. Dis.).

## Data Availability

The data information is placed in the supplementary files.
